# Nanohollow Carbon for Rechargeable Batteries: Ongoing Progresses and Challenges

**DOI:** 10.1007/s40820-020-00521-2

**Published:** 2020-09-19

**Authors:** Jiangmin Jiang, Guangdi Nie, Ping Nie, Zhiwei Li, Zhenghui Pan, Zongkui Kou, Hui Dou, Xiaogang Zhang, John Wang

**Affiliations:** 1 Jiangsu Key Laboratory of Electrochemical Energy Storage Technology, College of Material Science and Engineering, Nanjing University of Aeronautics and Astronautics, Nanjing, 210016 People’s Republic of China; 2 Department of Materials Science and Engineering, National University of Singapore, Singapore, 117574 Singapore; 3 Industrial Research Institute of Nonwovens and Technical Textiles, College of Textiles and Clothing, Qingdao University, Qingdao, 266071 People’s Republic of China; 4 Key Laboratory of Preparation and Application of Environmental Friendly Materials, College of Chemistry, Jilin Normal University, Siping, 136000 People’s Republic of China

**Keywords:** Hollow carbon nanospheres, Nanopolyhedrons and nanofibers, Template synthesis, Rechargeable batteries, Electrochemical performance

## Abstract

Among the various morphologies of carbon-based materials, hollow carbon nanostructures are of particular interest for energy storage. They have been widely investigated as electrode materials in different types of rechargeable batteries, owing to their high surface areas in association with the high surface-to-volume ratios, controllable pores and pore size distribution, high electrical conductivity, and excellent chemical and mechanical stability, which are beneficial for providing active sites, accelerating electrons/ions transfer, interacting with electrolytes, and giving rise to high specific capacity, rate capability, cycling ability, and overall electrochemical performance. In this overview, we look into the ongoing progresses that are being made with the nanohollow carbon materials, including nanospheres, nanopolyhedrons, and nanofibers, in relation to their applications in the main types of rechargeable batteries. The design and synthesis strategies for them and their electrochemical performance in rechargeable batteries, including lithium-ion batteries, sodium-ion batteries, potassium-ion batteries, and lithium–sulfur batteries are comprehensively reviewed and discussed, together with the challenges being faced and perspectives for them.

## Highlights

- The synthesis strategies of nanohollow carbon materials, including nanospheres, nanopolyhedrons, and nanofibers are summarized.
- Nanohollow carbon materials used as electrode materials in several types of rechargeable batteries are reviewed.
- The challenges being faced and perspectives of nanohollow carbon materials are discussed.

## Graphical abstract

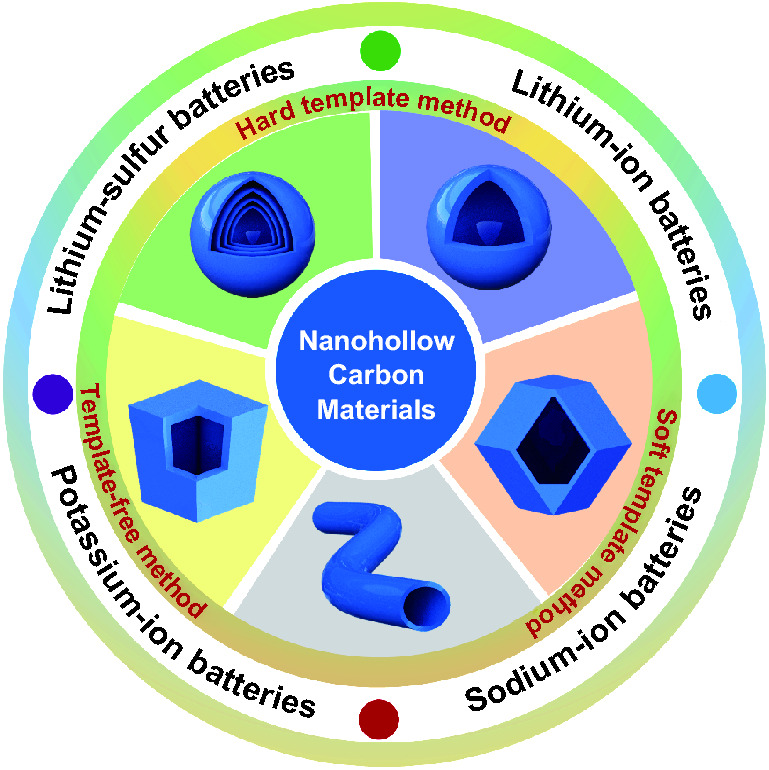

## Introduction

Carbon-based materials are among both the oldest and newest materials, in the entire human civilization of more than 5000 years, and the recent discoveries being fullerenes, carbon nanotubes (CNTs), and more recently graphene [1]. There is no doubt that these carbon-based materials are playing an irreplaceable part in our daily life, the ever-rapidly advancing technologies in the twenty-first century, and future scientific advances. In the past two decades, a series of carbon nanostructures have been developed, such as carbon dots, nanoparticles, nanorods, nanotubes, nanofibers, nanosheets, various core–shells, and nanohollow structures [2–9]. Among them, carbon hollow nanostructures of different morphologies represent a large group of carbon-based materials that have been tuned for high specific surface area in association with the high surface-to-volume ratios, controllable pores and pore size distribution, high electrical conductivity, variable crystallinities, and excellent chemical and mechanical stability [3, 5, 10–12]. By definition, these “nanohollows” refer to the various carbon nanostructures with an appropriate void nanospace distribution inside a distinct nanoshell, either relatively dense or porous, and their dimensions are in the nanometer scales. In morphology, they can be of nanospheres, nanopolyhedrons, and nanofibers.

**Fig. 1:**
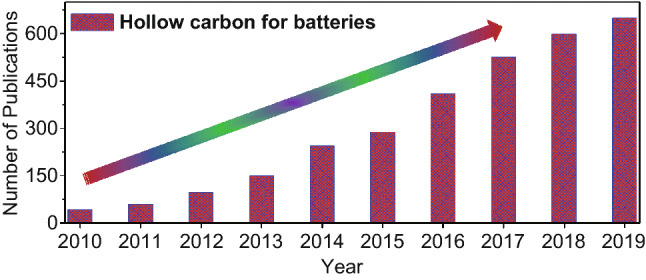
Number of publications searched by using “hollow carbon materials for batteries” on the Web of Science in the past 10 years

Since the 1990s, carbon-based materials have been widely employed as electrode materials in various energy storage and conversion devices, especially different types of rechargeable batteries, where graphite is the most widely used anode material for almost all commercial lithium-ion batteries until now [13]. Notably, these rechargeable batteries store charges by the Faraday reaction process and the corresponding electrochemical kinetics are relatively slow [14–16]. For example, carbon materials in any bulk form offer a limited population of active sites and require long ion diffusion pathways, leading to badly compromised reaction kinetics and poor performance. In view of this, carbon-based materials have been largely developed with unique nanostructures to improve the overall electrochemical performance, safety, and durability [17–22]. The development of nanohollow carbon materials (NHCMs) is an effective approach to address some of the bottleneck problems for batteries and other energy storage devices, where their advantages can be listed as follows: Firstly, NHCMs exhibit high surface-to-volume ratios and thus more active sites for charge storages, which would also be beneficial to the shortened electrons transfer/ions diffusion, improved interfacial contact with electrolyte and wettability, resulting in high specific capacity and excellent rate performance. Secondly, NHCMs possess favorable structural and mechanical stability, which can effectively suppress the volume expansion in the repeated long-term cycles of rechargeable batteries, leading to outstanding cycle stability. Thirdly, their morphologies and surface chemistry can be flexibly designed and regulated for different applications. With these appealing advantages, NHCMs have been widely explored as electrode materials in different types of batteries in recent years, which are evidenced by the yearly rising number of scientific publications on nanohollow carbon materials for batteries (Fig. 1).

To design and fabricate NHCMs with different morphologies, various templating and non-templating techniques have been exploited in the past two decades, including silica, polystyrene, calcium carbonate, surfactants, and copolymers for generating emulsion droplets, micelles, vesicles, etc. [2]. Among the various templating techniques, there are both soft and hard templates. For example, one of the early examples of hard templates was the use of hollow silica and inorganic–polymer hybrid nanospheres, reported by Caruso and co-workers in 1998 [23]. In parallel, various chemistry approaches have been taken to manipulate the intrinsic structure, defects, crystallinity, functional groups, and thus the resultant properties, notably by heteroatom (N, B, S, P, etc.) doping of NHCMs, which are aimed at improving the storage capacity and electrochemical kinetics in rechargeable batteries [24–26]. There are several recent reviews on carbon-based materials, such as carbon nanotubes and graphene, for applications in supercapacitors and batteries [27–31]. More recently, there are also a couple of reviews on carbon nanospheres and core–shell nanostructures for energy storage devices [32–35]. Nevertheless, there is a limited specific discussion in addressing NHCMs, especially with respect to their applications in rechargeable batteries, although there have been a rising number of studies, as mentioned above.

**Fig. 2:**
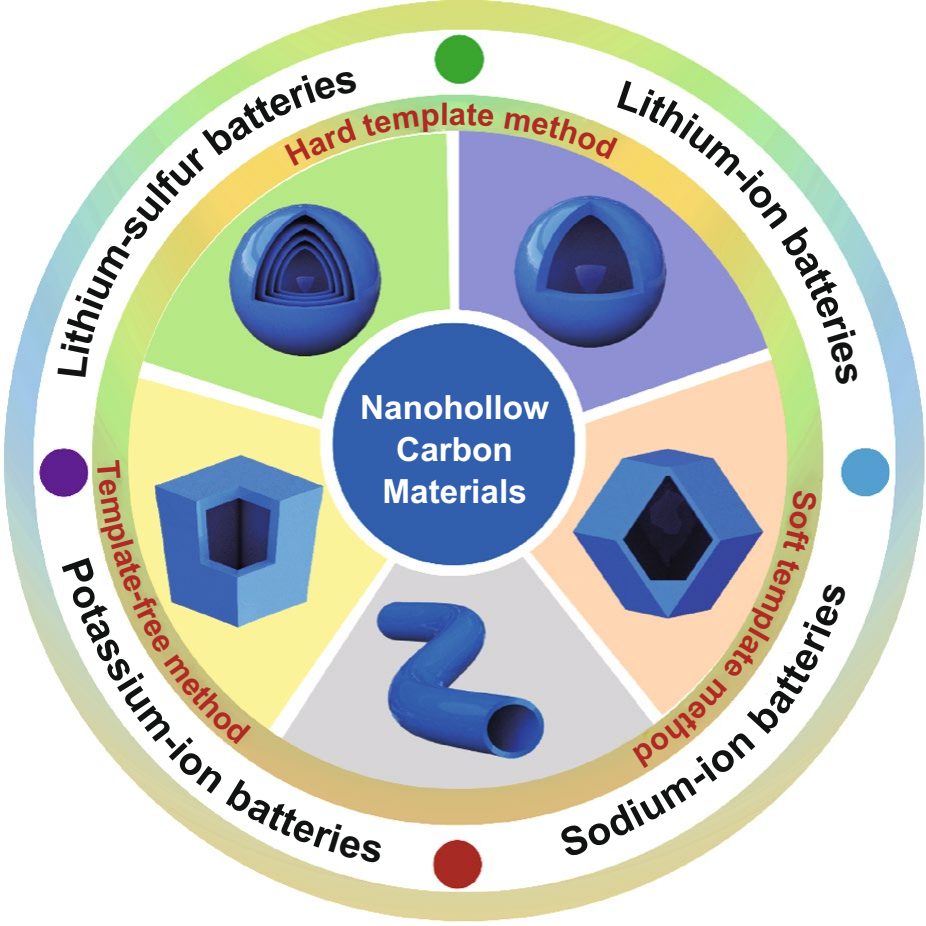
Schematic illustrations of various morphologies for nanohollow carbon materials used for rechargeable batteries

Herein, we will look into the ongoing progresses on the recent development of several key types of NHCMs, including hollow carbon nanospheres, nanopolyhedrons, and nanofibers, and their performances and applications in rechargeable batteries (Fig. 2). Firstly, the design and synthesis strategies of NHCMs through the hard templates, soft templates, and template-free approaches are carefully examined. Given the apparent advantages in structure and electrochemical performance of NHCMs, they have been widely employed as electrode materials for different types of rechargeable batteries, such as lithium-ion batteries (LIBs), sodium-ion batteries (SIBs), potassium-ion batteries (PIBs), and lithium–sulfur batteries (LSBs). There are also the challenges and future perspectives for NHCMs, in particular in connection with the rise of new generation energy storage devices in the coming few years.

## Controlled Formation of Nanohollow Carbon

Over the past two decades, nanohollow carbon materials (NHCMs) have been considered as an important class of carbon-based materials, and their preparation and performance in energy storage have become a research hot spot. More important than anything else is the controlled formation of nanohollow carbon. In this connection, the use of a pre-made nanostructural template is one of the most effective strategies toward achieving the designed nanohollow structure. Generally, the synthesis strategies for NHCMs can be classified into three major groups, including the hard templates, soft templates, and template-free approach. In parallel, there is a wide range of precursors that have been widely explored for these strategies. Among them, metal–organic frameworks (MOFs) have been studied as both templates and precursors more recently. For nanohollow carbon fibers, electrospinning has been most commonly used.

### Hard Template Methods

Hard templates are among the early approaches to prepare NHCMs, mainly for nanohollow carbon spheres, where the processes generally involve four steps: (1) design of a suitable rigid solid template, (2) coating the template with a carbon precursor, (3) high-temperature pyrolysis, and (4) removing the template. A series of hard templates have been utilized so far, including ceramic types such as silica, polymers such as polystyrene, inorganic salts, and even metallic particles.

**Fig. 3:**
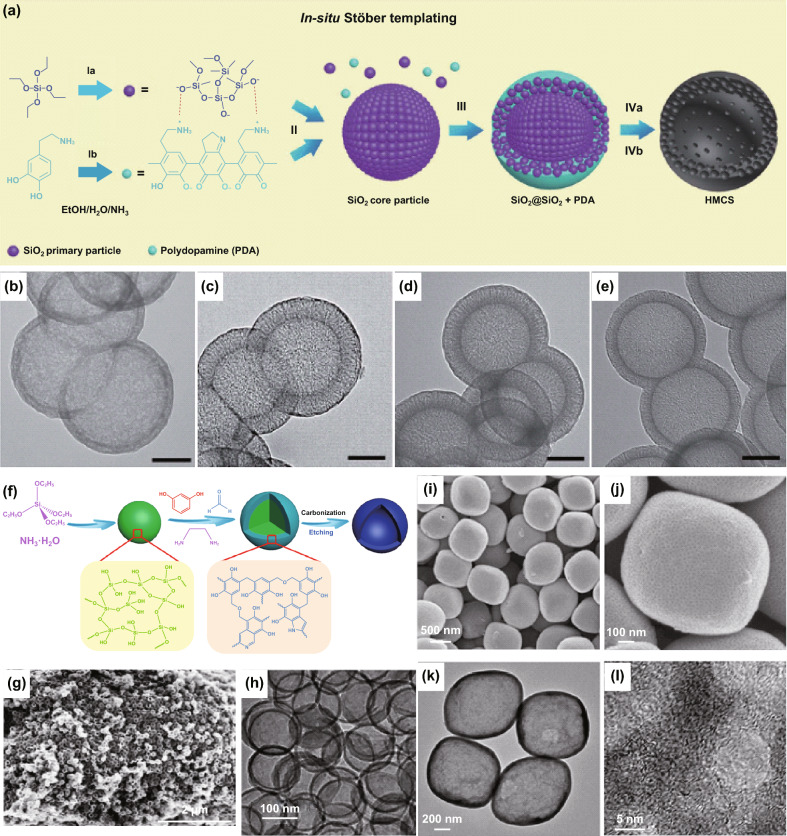
**a** Schematic illustration of the synthesis process of HMCS by using SiO_2_ as hard templates, **b–e** TEM images of the cavity and shell thickness of the as-fabricated HMCS, scale bars: 100 nm. Reproduced with permission from Ref. [36]. Copyright 2016, Royal Society of Chemistry. **f** Schematic illustration of the synthesis process of NC, **g** SEM, and **h** TEM images of NC. Reproduced with permission from Ref. [40]. Copyright 2017, Elsevier. **i**, **j** SEM and **k**, **l** TEM images of the carbon colloidosome shells. Reproduced with permission from Ref. [41]. Copyright 2016, Wiley–VCH

Silica (SiO_2_) nanospheres are the most widely used hard templates to synthesize NHCMs, owing to their tunable size from nanometers to micrometers, negative surface charge, relatively low cost, as well as good stability. They can be subsequently removed by etching using HF or hot NaOH solution, where the hollow nanostructure can be remained. For instance, Yu et al. designed hollow mesoporous carbon spheres (HMCS) derived from the silica–polydopamine (PDA) nanocomposite spheres by using SiO_2_ nanosphere templates [36]. The synthesis diagram of HMCS is shown in Fig. 3a, where the tetraethyl orthosilicate (TEOS) was first added to an EtOH/H_2_O/NH_3_ mixed solution to form SiO_2_ particles (2–3 nm). Notably, the suspension of the mixed solution could undergo secondary nucleation to form monodispersed SiO_2_ clusters. PDA was then added to the reaction system (after the addition of TEOS) for forming a SiO_2_@SiO_2_/PDA core–shell structure. The HMCS could then be obtained after carbonization and NaOH etching of the SiO_2_ templates. The as-synthesized NMCS exhibits a nanohollow spherical structure and controllable pore configuration, cavity size, and shell thickness. In particular, the shell thickness of NMCS can be controlled in the range of 15 to 55 nm, and the cavity diameters are in 285–162 nm, respectively (Fig. 3b–e). Noted that there are several carbon source options to achieve the nanohollow carbon spheres, including glucose [37], polypyrrole [38], ionic liquids [39], and so on. These carbon precursors lead to a stable nanohollow structure, when SiO_2_ is used as a template.

It should be pointed out that the resorcinol–formaldehyde (RF) has been commonly used as a fascinating and versatile carbon precursor. For example, Zhao et al. utilized the RF as the carbon precursor and ethylenediamine (EDA) by a sol–gel method to prepare the N-doped carbon hollow microspheres (NC) (Fig. 3f) [40]. The as-fabricated NC displayed a rather uniform size of ~ 120 nm with good mechanical strength (Fig. 3g). TEM studies revealed that the NC thus made exhibited hollow nanostructure with a thickness of ~ 10 nm (shell) and a diameter of ~ 100 nm (core) (Fig. 3h). Hyeon and co-workers developed nanohollow carbon capsules using phenol resin as the carbon sources and mesoporous SiO_2_ as the templates [42]. Fuertes et al. produced the SiO_2_@RF spheres with a core@shell structure [43], where the derived hollow carbon nanostructure is 150–500 nm in diameter. Zheng et al. applied a sol–gel process to synthesize the hollow carbon spheres with a high specific surface area of 1286 m^2^ g^−1^ [44]. Yu et al. reported a new surfactant-free sequential heterogeneous nucleation pathway using the monodispersed SiO_2_@RF@SiO_2_@RF composite to prepare mesostructured nanohollow carbon particles [45].

In addition to the sol–gel processes, SiO_2_ templates have been employed in other processes as the hard templates, such as chemical self-assembly, chemical vapor deposition (CVD), hydrothermal, and solvothermal approaches. For example, Lou et al. developed a chemically assisted strategy to obtain hollow carbon colloidosomes on various types of functional particles [41]. Their particle sizes can be tuned by size selection of the SiO_2_ nanosphere templates. As shown in Fig. 3i–l, the as-fabricated hollow carbon colloidosomes exhibit a cavity size of ~ 400 nm. CVD is an efficient and controllable process to prepare carbon-based materials. Zhao et al. prepared hollow carbon spheres (HCSs) by a CVD method using benzene as the carbon precursor and SiO_2_ spheres as the templates [46]. The HCSs can be controlled in either smooth single shells, or deformed single shells, or double shells, and N-doped shells. Chen and co-works also investigated the preparation of hollow carbon materials using the SiO_2_ template by CVD method [47, 48]. Titirici et al. proposed a hydrothermal carbonization process to achieve hierarchical hollow carbon materials [49], where some functional groups could be anchored on their surfaces under the hydrothermal conditions.

Many other hard templates have been utilized to synthesize nanohollow carbon materials. The surface charges of polystyrene nanospheres (PS) are negative, which is also a class of widely used hard template [50–52]. For instance, Lu and colleagues proposed a confined nanospace pyrolysis process to produce uniform hollow carbon nanospheres (HCSs), by consecutive surface coating on the PS templates [53]. Porous N-doped hollow carbon spheres (PNHCSs) have been prepared using polyaniline as the carbon sources and PS as the templates by Dou et al. [54]. Calcium carbonate (CaCO_3_) is also an effective hard template for producing NHCMs [55–57]. Our groups developed the N-doped hollow porous carbon spheres (NPCSs) by using CaCO_3_ spheres as the templates and polydopamine as the carbon source [58]. Notably, the CaCO_3_ nanosphere can be used as both a hard template and an activator in the high-temperature pyrolysis; thus, the as-prepared NPCS exhibits a high specific surface area of 1984 m^2^ g^−1^. Besides, some metallic particles have also been proposed as hard templates, such as the metallic Mg [59, 60], Na [61], and Zn [62–64].

### Soft Template Methods

Although hard templates have been widely employed in the preparation of NHCMs giving rise to different nanostructures, certain issues exist for them. For example, the hard templates have to be pre-made, which can be a tedious process and add on the overall cost. The processes using hard templates are typically multi-steps and time-consuming, due to the preparation of precursor materials and their coatings, high-temperature carbonization, and the subsequent templates removal by dissolving them in strong acids or alkaline conditions, which are not environmentally friendly. In this regard, soft template methods are more attractive, because the applied templates can either be converted into carbon or be removed in the same carbonization process. Some commonly used soft templates include copolymers and surfactants, which generate the required emulsion droplets, micelles and vesicles, and gas bubbles.

**Fig. 4:**
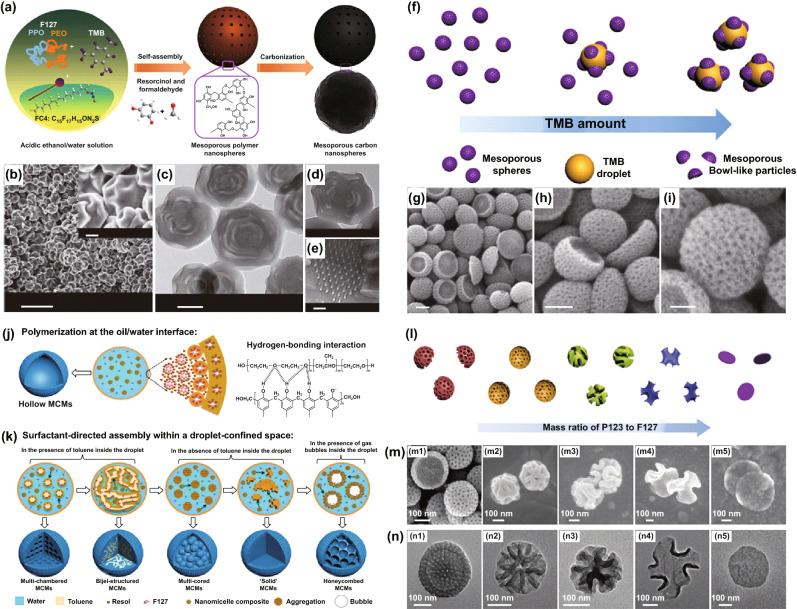
**a** Schematic illustration of the formation process of MCNs. **b** SEM images, **c**, **d** TEM images, and **e** HRTEM image of MCNs. Reproduced with permission from Ref. [65]. Copyright 2013, Nature Publishing Group. **f** Schematic representation of the mesoporous PDA particle formation. **g–i** FESEM images of the as-synthesized bowl-like hollow carbon particles. Reproduced with permission from Ref. [66]. Copyright 2016, American Chemical Society. Schematic illustration of **j** interfacial polymerization of phenolic resol in the presence of Pluronic F127, and **k** evolution of the interior structures. Reproduced with permission from Ref. [67]. Copyright 2018, Wiley–VCH. **l** Schematic representation, **m** FESEM (top), and **n** TEM (bottom) images of mesophase transition PDA particles with different mass ratios of P123 to F123. Reproduced with permission from Ref. [68]. Copyright 2018, Wiley–VCH. Scale bars are **b** 1 mm (inset image: 100 nm), **c** 200 nm, **d**, **g**, **h** 100 nm, **e** 10 nm, and **i** 50 nm

Although various soft templates have been studied and several successful examples have been documented, in general, the successful synthesis of mesoporous or hollow carbon remains a challenge, especially at large scale, due to the relatively weak self-assembly ability of certain precursor components and the likely cross-linking of neighboring building blocks. For example, Qiao et al. used the cationic fluorocarbon surfactant FC4 (C_3_F_7_O(CFCF_3_CF_2_O)_2_CFCF_3_CONH(CH_2_)_3_N^+^(C_2_H_5_)_2_CH_3_I^−1^) and triblock copolymer Pluronic F127, EO_106_PO_70_EO_106_ (EO, ethylene oxide; PO, propylene oxide) as the soft templates, together with RF as the carbon precursors to prepare mesoporous carbon nanospheres (MCNs) (as shown in Fig. 4a) [65]. By adjusting the key reaction parameters, multi-layered mesoporous RF hollow nanospheres can be synthesized, which can be converted into different hollow MCNs (Fig. 4b–e). Notably, the co-templating of FC4 and Pluronic F127 and the cross-linking properties of the RF precursors, which showed a large degree of shrinkage during the high-temperature pyrolysis process, lead to the formation of hollow or mesoporous carbon nanostructures. Lou et al. proposed the mixed liquids of 1,3,5-trimethylbenzene (TMB) and water with emulsion droplets as the soft template [66]. The bowl-like hollow PDA particles were synthesized through an emulsion-induced interface anisotropic self-assembly by using the purposely designed TMB soft template and PDA polymer (Fig. 4f). As shown in Fig. 4g–i, the as-derived hollow mesoporous carbon remained a bowl-shaped hollow morphology, with a specific surface area of 619 m^2^ g^−1^.

Yang et al. also proposed the use of water droplets in nanoparticle-stabilized emulsions (Pickering emulsions) as the soft templates [67]. The interior-structured mesoporous carbon microspheres (MCMs) could be successfully obtained by the surfactant assembly within the pickering emulsion droplets templates, and the phenolic resole oligomers were used as the carbon precursors. In particular, the phenolic resole oligomers were co-assembled with Pluronic F127 molecules via hydrogen bonding interactions, and the formation of the nanomicelle-type composite induced a new oil/water interface, leading to a mesoporous structure at around the inner surface of the water droplet (Fig. 4j). The interior structures of MCMs thus derived (such as hollow, multi-chambered, bijel-structured multi-cored “solid,” and honeycombed) could be regulated by tailoring the concentration of nanomicelles with water droplets (Fig. 4k). Similarly, Ji et al. produced N-doped hollow nanospheres (N-NHCM) through the carbonization of hollow ZIF-8 nanospheres, which were prepared by an emulsion-based interfacial reaction [69]. In addition, Yao and co-workers presented an approach of using o-phenylenediamine (oPD) oligomers as the soft templates, by hydrogen bonding to form polymer microspheres [70], which could be transformed into nitrogen and oxygen co-doped hollow carbon spheres (HCSs) upon high-temperature pyrolysis process. Subsequently, Ye et al. applied an O/W/O inverse-emulsion system as the soft template and RF as the carbon precursor to develop hollow carbon particles [71].

While the soft templates can lead to mesoporous hollow materials, a precision regulation in the pore configuration and feature remains a challenge in several studied systems. In this regard, Lou et al. proposed a novel dual-soft-template approach to prepare walnut-shaped macro/mesoporous PDA particles with bicontinuous channels ranging from 20 to 95 nm [68]. A mixture of two similar block copolymers (P123 and F127) was employed as the dual soft templates in their research. Note that the mass ratio of P123 to F127 has a great impact on the mesophase transition for the formation of mesostructured PDA particles (Fig. 4l). As shown in Fig. 4m, n, the walnut-shaped PDA particles could be carbonized into hollow mesoporous carbon particles with morphology and pore structure largely unchanged. Besides, some common surfactants could also be used as the soft templates to prepare nanohollow carbon materials [72]. For instance, Li and Zhang et al. utilized the anionic surfactant sodium dodecyl sulfate (SDS) as the soft template to synthesize hollow carbonaceous capsules and hollow carbon nanospheres, respectively [73, 74]. Tashima and co-workers proposed the cetyltrimethylammonium bromide (CTAB) as the soft template and 1,3,5-trimethylbenzene (TMB) and tertbutanol (t-BuOH) as the co-surfactants to prepare nanohollow carbon materials [75, 76].

### Template-Free Methods

Although both the hard template and soft template methods have been widely employed in the synthesis of nanohollow carbon materials up to now, the hard template strategies are typically multi-steps and time-consuming owing to the need for preparation and subsequent removal of precursor template materials. It is also difficult to accurately tune the hollow morphologies and components of NHCMs by using the soft template strategies. To this end, the template-free approach is a facile and low cost for synthesizing NHCMs.

**Fig. 5:**
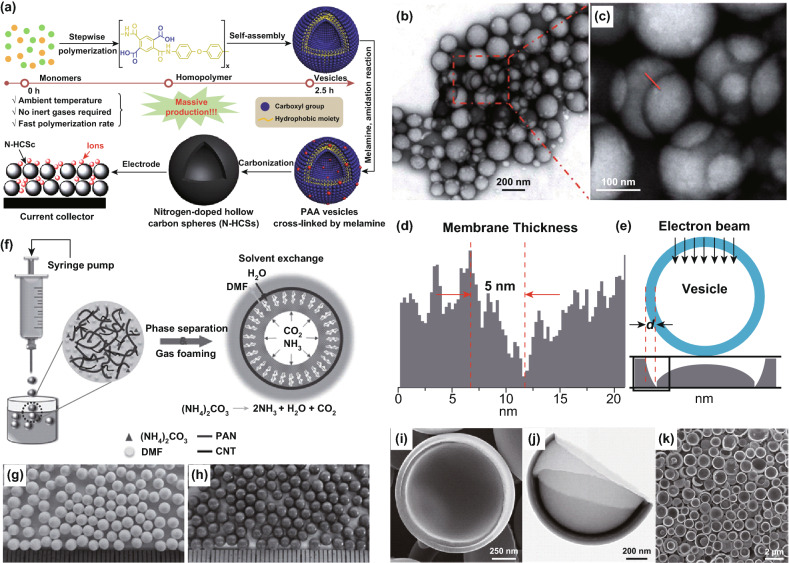
**a** Formation of N-HCSs by homopolymers, self-assembly, and carbonization processes. **b**, **c** TEM images of PAA vesicles with some stack-up vesicles. **d** Electron transmittance chart related to the red scan line in **b**, **e** Electron transmittance simulation of hollow spheres. Reproduced with permission from Ref. [77]. Copyright 2016, Royal Society of Chemistry. **f** Schematic illustration of the preparation of hollow polymer spheres. Photographs of **g** hollow polymeric PAN spheres, and **h** CNTs-reinforced hollow polymeric spheres. Reproduced with permission from Ref. [82]. Copyright 2016, Wiley–VCH. **i** SEM and **j** TEM images of the porous carbon double-hemispheres. **k** Low-magnification SEM image of a monolayer of the carbon double-hemispheres. Reproduced with permission from Ref. [83]. Copyright 2012, Wiley–VCH

In this connection, certain hollow polymers/precursors can be prepared by self-polymerization or self-assembling, and then, a high-temperature pyrolysis is carried out to overt them into NHCMs. For example, Wu and co-workers synthesized the amphiphilic homopolymer (PAA) through a polymerization process without any templates, which could be self-assembled into rather uniform vesicles by directly adding water to the PAA solution without any purification (Fig. 5a) [77]. As shown in Fig. 5b, c, the diameter of the as-assembled PPA vesicles was ~ 200 nm. By the electron transmittance chart and electron transmittance simulation, the membrane thickness of PAA vesicles was ~ 5 nm (Fig. 5d, e). Nitrogen-doped hollow carbon spheres (N-HCSs) can then be prepared by the carbonization of the PAA vesicles cross-linked by melamine. Authors group also developed a controllable solvothermal route to design the N-doped hollow carbon microspheres (NHCMs), which were formed by the self-assembly of hierarchical polyimide nanosheets by controlling of a suitable polymerization time and solvent without any additional catalyst and template [78]. The as-fabricated NHCM displayed a hierarchical porous structure with a high specific area of 1005 m^2^ g^−1^. Notably, the template-free method for fabricating NHCM is environment friendly and does not require the tedious processes of adding and removing any templates. For example, Wu and Niwase et al. prepared hollow carbon materials through heat treatment of the resole modified with poly(ethylene glycol) monomethyl ether (resole-PEG), with C60 fullerene powder as the polymeric carbon precursor [79, 80]. Hui et al. utilized the solid melamine–formaldehyde resin spheres to develop nitrogen- and oxygen-co-doped hollow carbon spheres (HCSs) by high-temperature pyrolysis [81].

Self-assembly provides a promising pathway for the synthesis of carbon-based materials with nanohollow structures. Long et al. reported hollow polyacrylonitrile (PAN) spheres by a gas-foaming-assisted phase-inversion process, where the liquid–liquid phase-inversion process and a self-assembly process were coupled (Fig. 5f) [82]. As shown in Fig. 5g, the PAN spheres thus made showed robust and hollow architectures, and their sizes could be tailored by changing the needle sizes and the temperatures of the water bath. The mechanical properties of the hollow composite-type spheres could be enhanced through adding CNTs in the precursor solution (Fig. 5h). Hollow carbon spheres (HCSs) were then obtained by the carbonization of the oxidized PAN spheres by a high-temperature treatment. Similarly, spray pyrolysis has been considered as an effective to prepare nanohollow carbon materials [84, 85]. For instance, S. Suslick et al. proposed an ultrasonic spray pyrolysis to synthesize porous carbon materials with alkali propiolates (HC ≡ CCO_2_M, M = Li, Na, and K) as the carbon precursors [83]. As shown in Fig. 5i–k, different structures and morphologies of hollow carbon materials can be formed by mixing the alkali propiolates with different mole ratios. A one-step aerosol process to synthesize hollow carbon nanocapsules has been reported by Lu and co-workers, who employed an enzymatically polymerized poly(4-ethylphenol) as the carbon precursor [86]. NHCMs can be also formed through a proper plasma process. For example, Kim and Charlier et al. proposed an electric plasma discharge and thermal plasma process to prepare hollow carbon materials [87, 88]. García and co-workers presented a study on the chlorination of bis(benzene)chromium (Cr(C_6_H_6_)_2_) to achieve the hollow and solid carbon spheres at two reaction temperatures [89]. The as-fabricated hollow carbon spheres exhibited a high surface area of 1761 m^2^ g^−1^.

### MOFs-Derived Hollow Carbon Nanopolyhedrons

Metal–organic frameworks (MOFs) are crystalline coordination compounds composed of metal ions (or metal clusters) and organic ligands, which have attracted much attention both scientifically and technologically [90]. Due to their tunable chemical compositions, morphologies, level of porosity, international pore configuration, and surface functionalities that can be regulated by the combinations of organic and metal/inorganic constituents, they have been widely applied in adsorption, gas storage, catalysis, drug delivery, energy storage, and conversion [91]. In addition, MOFs have been demonstrated as excellent precursors to prepare various porous materials, including those carbon-based, as reported by Xu and co-workers in 2008 [92]. A subsequent report on MOFs-derived porous carbon was made by Yamauchi et al. in 2012 [93]. Since then, MOFs-derived carbons have emerged as a large class of porous materials studied for various applications [3, 94, 95]. Nevertheless, the common challenges for these MOFs-derived carbon materials are related to the carbonization process, such as the undesired pore structure formed, aggregation of metal/nonmetal particles, and poor control in structural evolution [20]. To address some of these issues MOFs themselves can act certain templating roles, in addition to being the precursors. Therefore, considerable efforts have been made in the tuning of various processing conditions to form the designed MOFs in the first place, and then the condition to convert them into the desired hollow/porous carbon-based structures. Similarly, there has been considerable progress made with both the hard and soft templating of MOFs and MOF-derived carbon-based materials. In this connection, some of the governing principles that have been discussed above on the hard and soft templating approaches would be applicable to them.

**Fig. 6:**
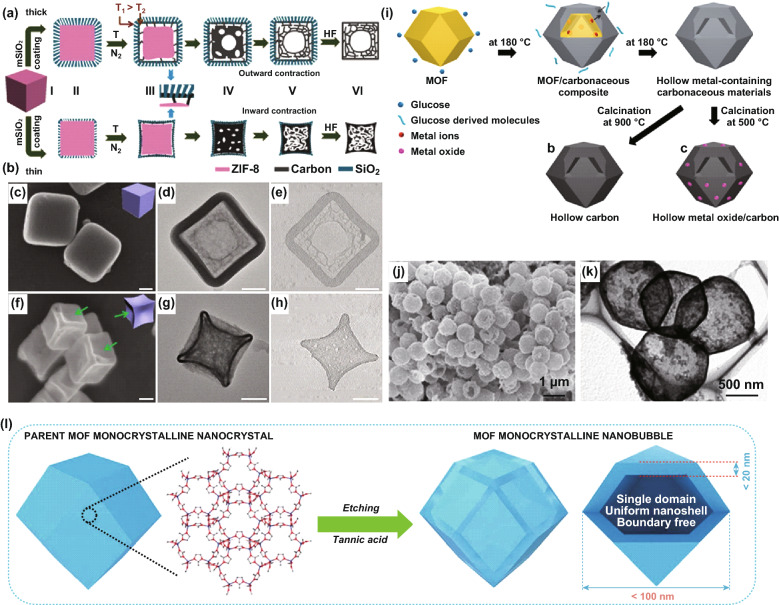
Schematic illustration of the fabrication processes of **a** HMCNCs and **b** SMCNCs. **c**, **f** SEM images, **d**, **g** TEM images, and **e**, **h** electron tomography slices of carbon@mSiO_2_-40 and carbon@mSiO_2_-8, respectively, scale bar: 100 nm. Reproduced with permission from Ref. [96]. Copyright 2018, Wiley–VCH. **i** Schematic illustration of the preparation of hollow carbonaceous materials. **j** SEM, and **k** TEM images of the HZC-2.5 M-2 h. Reproduced with permission from Ref. [100]. Copyright 2018, American Chemical Society. **l** Schematic illustration of the spatially controlled etching to produce monocrystalline ZIF nanobubbles. Reproduced with permission from Ref. [101]. Copyright 2017, Royal Society of Chemistry

As a main elemental component in MOFs is carbon, carbon-based materials can be derived by carbonization of MOFs without adding other precursors. However, due to the rather complicated steps involved in the actual conversion process, there is need to control the processing conditions applied to the carbonization process, such as temperature, atmosphere/medium, and even time. In the process, there are steady changes in the chemical composition, types of phases, both internal pore structure and surface conditions, as well as morphology. A few MOFs can be converted into hollow carbon structure, by carefully choosing the MOF types and experimental conditions. To tailor the desired hollow carbon structure, certain templating approach is shown to be useful. For example, in a stress-induced orientation contraction approach, Ye and co-workers used ZIF-8 nanotubes as the precursor and then coated them with a thick and thin mesoporous silica layer (mSiO_2_), respectively. Mesoporous hollow carbon nanotubes (HMCNCs) and solid mesoporous carbon nanotubes (SMCNCs) without hollow cavity could then be obtained after pyrolysis and acid treatment (Fig. 6a, b) [96]. The ZIF-8 nanotubes would have contracted strongly during the high-temperature pyrolysis process, and the carbonization occurred preferentially at the interface between ZIF-8 and mSiO_2_ coating, where the thick mSiO_2_ layer was rigid enough to counteract the inward contraction, but not the thin mSiO_2_ layer, so the respective HMCNCs and SMCNCs were formed by the different mSiO_2_ thicknesses. The SEM and TEM images of the HMCNCs (the thickness of mSiO_2_ was about 40 nm, Fig. 6c–e) and SMCNCs (the thickness of mSiO_2_ was about 8 nm, Fig. 6f–h) further explain that the rigid-interface-induced outward contraction by the mSiO_2_ layer thickness was effective to regulate ZIF-8 derived carbon structure. Besides, the mesoporous silica (mSiO_2_) had been coated on the Co-based MOFs (ZIF-67) to synthesize 3D hollow carbon materials, together with carbon nanotubes on their surface by the Co catalysis during the pyrolysis process [97]. Similarly, carbon nanotube-decorated N-doped hollow carbon can be prepared through pyrolysis of SiO_2_ and bimetallic ZIFs-Co_*x*_Zn_1−*x*_ based on a mixed ions strategy [98]. Hollow N-doped carbon can be also achieved using ZIF-8 as the cores and polydopamine (PDA) as the shells via heat treatment by the stresses-induced orientation contraction [99].

The synthesis of MOFs-derived hollow carbon nanopolyhedrons has been successfully demonstrated through SiO_2_ coating, carbonization, and etching process, whereas this procedure requires a uniform coating around the high-curvature surface and post-treatment to remove the template, which is rather complicated and time-consuming. Yamauchi and co-workers had proposed that ZIF-8 could be used to fabricate hollow carbonaceous composites through a hydrothermal reaction with glucose (Fig. 6i), where the acid generated from the hydrolysis of glucose led to the decomposition of ZIF-8 [100]. As shown in Fig. 6j, k, ZIF-8-derived hollow carbonaceous composites can be transformed into hollow carbon and ZnO/C nanocomposites after pyrolysis at 900 and 500 °C, respectively. They have also developed a spatially controlled tannic acid solution etching strategy to prepare monocrystalline ZIF nanobubbles (Fig. 6l), which can be converted into hollow carbon nanobubbles under an optimal pyrolytic condition [101]. Furthermore, Wang et al. found that the phytic acid (PA) can slowly etch ZIF-67, forming a hollow nanostructure [102]. More recently, a series of MOFs-derived hollow carbon nanopolyhedrons have been reported by using MOFs as the cores (structural templates) and various polymers as the shells achieved through high-temperature pyrolysis [103]. The polymer coating layers, such as poly(cyclotriphospazene-co-4,4′-sulfonyldiphenol) (PZS) [104, 105], oligo(cyclotriphosphazene-co-hexahydroxytriphenylene) (OCHT) [106], and resorcinol–formaldehyde polymers [107], have been demonstrated to be feasible.

### Electrospinning of Hollow Carbon Nanofibers

One-dimensional (1D) hollow carbon nanofibers have been exploited for energy storage and conversion, owing to their large length to diameter ratio and high surface area, which can provide charge storage sites and fast pathways for electron transport [3]. These 1D hollow carbon nanofibers also show robust mechanical flexibility, which can be easily grown on to substrate supports to form self-standing flexible electrodes for energy storage devices. Up to now, there are several strategies to prepare the 1D carbon nanofibers, such as electrospinning, template synthesis, chemical vapor deposition, hydrothermal growth, and self-assembly. Among them, electrospinning is considered as the most facile and highly controllable approach to achieve the designed carbon nanofibers with hollow nanoarchitectures and freestanding functionality, including those uniaxial, co-axial, and triple-co-axial ones.

**Fig. 7:**
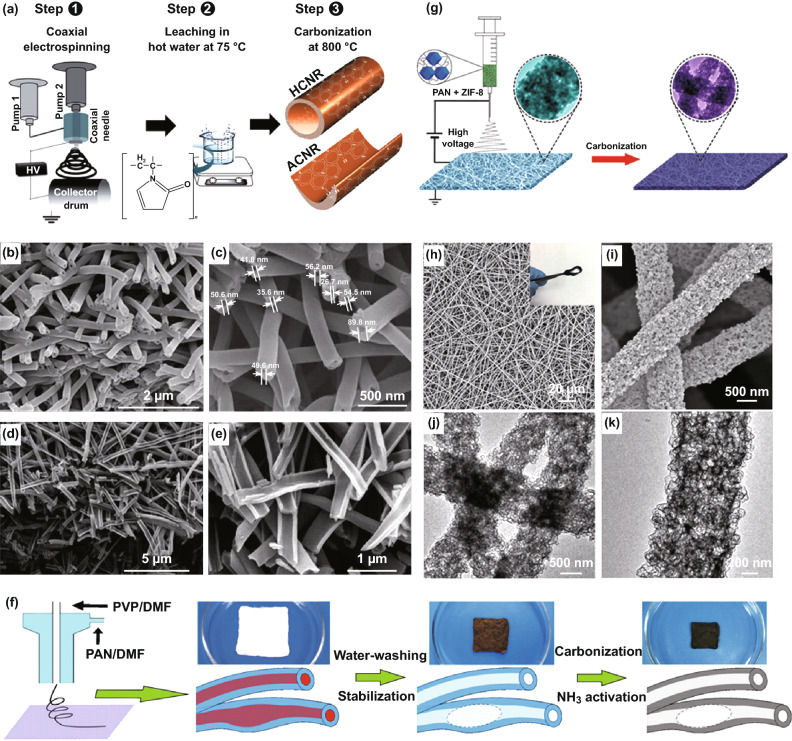
**a** Schematic illustration of the synthesis of HCNR and ACNR samples by co-axial electrospinning. SEM images of **b**, **c** HCNR, and **d**, **e** ACNR, respectively. Reproduced with permission from Ref. [108]. Copyright 2015, Royal Society of Chemistry. **f** Schematic illustration of the fabricating process of HCNF. Reproduced with permission from Ref. [109]. Copyright 2015, Elsevier. **g** Schematic illustration of the synthesis of HPCNFs-N. **h**, **i** SEM, and **j**, **k** TEM images of the HPCNFs-N. Reproduced with permission from Ref. [117]. Copyright 2017, Royal Society of Chemistry

Due to the high carbon yield and predictable mechanical strength of the resultant products, polyacrylonitrile (PAN) has been widely employed as the carbon precursor for electrospun carbon nanofibers, where co-axial electrospinning is the commonly used method for manufacturing 1D hollow carbon nanofibers. For example, Shanmugam and co-workers prepared hollow (HCNR)- and arch (ACNR)-shaped carbon nanotubes by the co-axial electrospinning (Fig. 7a), where PAN was used as the carbon precursor and polyvinyl pyrrolidone (PVP) was used as the sacrificial polymer with different flow rates [108]. As shown in Fig. 7b–e, the as-fabricated HCNR and ACNR nanostructures showed average diameters of 180 and 155 nm, where the core diameter was about 40–70 nm for the HCNR sample. Similarly, N-doped hollow carbon nanofibers (HACNFs) have been prepared via the co-axial electrospinning using PVP as the core precursor and PAN as the shell precursor, together with NH_3_ activation treatment (Fig. 7f) [109]. The as-obtained HACNFs exhibited an outer and an inner fiber diameter of ~ 300 and ~ 150 nm, as well as a high specific surface area of 655 m^2^ g^−1^. Up to now, different internal and external polymer precursor components have been developed for preparing hollow carbon nanofibers by the co-axial electrospinning, including a mixture of PAN/PVP as the shell and PVP as the core precursor [110], poly(styrene-co-acrylonitrile) (SAN) as the core and PAN/PVP mixture as the shell precursor [111], SAN solution as the core and polyacrylic acid (PAA) as the shell precursor [112], poly (methyl methacrylate) (PMMA) as the core precursor and PAN/PMMA mixture as the outer shell precursor [113], silicone oil as the inner core and PAN as the outside layer precursor [114], and so on [115, 116].

1D hollow carbon nanofibers can also be prepared by uniaxial electrospinning technique, which needs some additional materials to assist the synthesis. For instance, the hollow particle-based N-doped carbon nanofibers (HPCNFs-N) have been prepared by the carbonization of PAN/ZIF-8 composite nanofibers (Fig. 7g), where the ultrafine ZIF-8 nanoparticles were embedded into electrospun PAN precursor [117]. The primary ZIF-8 nanoparticles could be transformed into interconnected N-doped carbon hollow nanoparticles by high-temperature carbonization. As shown in Fig. 7h–k, the as-fabricated HPCNFs-N exhibited good flexibility, which consisted of numerous hollow nanoparticles interconnected with each other. Owing to such hierarchical porous structure (417.9 m^2^ g^−1^) and high N-doping level (7.85%), the HPCNFs-N showed remarkable specific capacitances and excellent cycle stability, when used as electrodes in supercapacitors. Similarly, Wang et al. also reported the preparation of N-doped hollow hierarchical carbon fibers (NCPFs) via electrospinning and further carbonization of the ZIF-8/PAN nanofibers [118]. The hard/soft templates materials were added to the electrospinning polymer solution for achieving the hollow carbon nanofibers. For example, Cui and co-workers used SiO_2_ as the template and pore-forming agent to prepare hollow bamboo-like carbon nanofibers [119]. Other hard templates included ZnO [120], SnO_2_/Fe_2_O_3_ [121], and PS microspheres [122]. Several soft templates have been also proposed to obtain hollow carbon nanofibers [123], together with triple-co-axial electrospinning, nozzle-less electrospinning, and other approaches [124–126].

## Application in Rechargeable Batteries

Lithium-ion batteries (LIBs), sodium-ion batteries (SIBs), potassium-ion batteries (PIBs), and lithium–sulfur batteries (LSBs) are among the rechargeable batteries. The electrochemical performances of these battery systems are largely determined by the electrode materials, and thus, the development of a superior electrode material plays an important role in these rechargeable batteries. Indeed, nanohollow carbon materials (NHCMs) have been widely investigated as the electrode materials; in particular, those hollow carbon nanospheres exhibit high surface-to-volume ratios, encapsulation capability, together with outstanding electrochemical performance when used in batteries, especially using as conductive host materials offer to inhibit the polysulfide entrapping and buffer the volume expansion in lithium–sulfur batteries. However, owing to nanohollow carbon spheres having the lower tap density and the larger cavity volume than some of hollow carbon materials, they give rise to a relatively lower volume energy density in the as-assembled batteries. In addition, the structural stability of hollow carbon nanospheres needs to be further improved during the repeated cycles. MOF-derived carbon nanopolyhedrons possess high specific surface areas and tailorable levels of porosity, which would aid to provide a path way for electrons to move and shorten the length of transfer channel. Nevertheless, the production yield of MOF-derived carbon nanopolyhedrons is low; this is a torturous problem for large-scale application in batteries. Hollow carbon nanofibers afford a high surface-to-volume ratio and a short transport pathway for ions, and they also show favorable opportunities in flexible battery devices, owing to their unique 1D morphology and outstanding flexibility. Even so, the reactive sites exposed by nanohollow carbon nanofibers are relatively limited, and thus, a combination of multiple activation strategies has often been employed when used in batteries. Even so, the reactive sites exposed by the nanohollow carbon nanofibers are relatively limited in number. Thus, an efficient combination of multiple entities would be of value for applications in batteries. For example, rechargeable batteries making use of a combination of different types of nanohollow carbons as the electrode materials can give rise to a high specific capacity, rate capability, and cycle stability. Some of the application examples are listed in Table 1, where they are compared with those with graphite and conventional carbon materials.

**Table 1:** Overall electrochemical performances of rechargeable batteries making use of nanohollow carbons, graphite, and hard carbon materials

Batteries	Materials	Highest capacity (mAh g^−1^)	Rate capability (mAh g^−1^)	Cycle numbers	References
LIBs	N-doped hollow CNT–CNF	1150 at 0.1 A g^−1^	320 at 8 A g^−1^	3500	[127]
LIBs	N-doped hollow carbon nanoflowers	528 at 2 C	298 at 10 C	1000	[128]
LIBs	N-doped double-shelled hollow carbon spheres	920.3 at 0.1 A g^−1^	292.9 at 5 A g^−1^	300	[129]
LIBs	Macro–mesoporous hollow carbon spheres	530 at 2.5 A g^−1^	180 at 60 A g^−1^	1000	[130]
LIBs	Carbohydrate-derived hollow carbon spheres	~ 400 at 1 C	~ 100 at 2 C	200	[131]
LIBs	Hollow carbon nanoparticles	863 at 0.1 A g^−1^	171 at 6.4 A g^−1^	200	[132]
LIBs	N-doped mesoporous carbon hollow spheres	485 at 0.5 A g^−1^	214 at 4 A g^−1^	1100	[133]
LIBs	Graphitic hollow carbon spheres	2007 at 0.1 A g^−1^	410 at 5 A g^−1^	1000	[134]
LIBs	Self-healing core–shell hollow carbon fibers	603.9 at 1 A g^−1^	499 at 2 A g^−1^	1500	[135]
LIBs	N and O co-doped hollow carbon spheres	1395 at 0.1 A g^−1^	606 at 5 A g^−1^	600	[136]
LIBs	Hollow carbon nanotubes	517.7 at 0.05 A g^−1^	436.4 at 0.2 A g^−1^	2000	[137]
LIBs	N-doped hollow carbon fibers	~ 600 at 0.5 C	~ 200 at 50 C	500	[138]
LIBs	Graphite	393 at 0.1 C	~ 120 at 10 C	800	[139]
LIBs	Holey graphite	425.7 at 0.1 C	95.7 at 2 C	500	[140]
LIBs	Natural graphite	395.6 at 0.1 C	246 at 50 C	200	[141]
SIBs	Multi-shelled hollow hard carbon nanospheres	360 at 0.03 A g^−1^	200 at 0.6 A g^−1^	150	[142]
SIBs	Hollow carbon nanospheres	160 at 0.2 A g^−1^	~ 50 at 10 A g^−1^	100	[143]
SIBs	P-doped hollow carbon sphere	234 at 0.1 A g^−1^	129 at 1.5 A g^−1^	300	[144]
SIBs	S/N-co-doped hollow carbon spheres	185 at 0.5 A g^−1^	110 at 10 A g^−1^	2000	[145]
SIBs	N-containing hollow carbon microspheres	296 at 0.2 A g^−1^	114 at 10 A g^−1^	1200	[146]
SIBs	Hollow carbon nanowires	251 at 0.05 A g^−1^	149 at 0.5A g^−1^	500	[147]
SIBs	Hollow carbon nanofibers	326 at 0.02 A g^−1^	85 at 1.6 A g^−1^	5000	[148]
SIBs	N/P co-doped hollow carbon nanofibers	358 at 0.05 A g^−1^	140 at 5 A g^−1^	2000	[149]
SIBs	N/S dual-doped hollow carbon fibers	264 at 0.1 A g^−1^	64 at 10 A g^−1^	4000	[150]
SIBs	Polyhedral-shaped hollow porous carbon	227 at 1 A g^−1^	133 at 20 A g^−1^	9000	[151]
SIBs	3D hollow reticulate hard carbon	160 at 0.05 A g^−1^	50 at 2 A g^−1^	1000	[152]
SIBs	Hard carbon	430.5 at 0.03 A g^−1^	~ 50 at 2 A g^−1^	200	[153]
SIBs	Orange peel-derived hard carbon	180 at 0.015 A g^−1^	~ 120 at 0.14 A g^−1^	100	[154]
SIBs	Defective hard carbon	~ 300 at 0.1 A g^−1^	~ 125 at 2 A g^−1^	200	[155]
PIBs	Sulfur-grafted hollow carbon spheres	581 at 0.025 A g^−1^	110 at 5 A g^−1^	1000	[156]
PIBs	Activated hollow carbon nanospheres	370.2 at 0.2 A g^−1^	137 at 4 A g^−1^	5000	[157]
PIBs	N-doped hollow carbon nanospheres	326 at 0.05 A g^−1^	141 at 2 A g^−1^	2500	[158]
PIBs	Hollow interconnected neuron-like carbon	340 at 0.1 C	~ 100 at 2 C	500	[159]
PIBs	Hollow multihole carbon bowls	304 at 0.1 A g^−1^	182 at 2 A g^−1^	1000	[160]
PIBs	Soft carbon semi-hollow microrods	314 at 0.1 A g^−1^	~ 100 at 1 A g^−1^	500	[161]
PIBs	Graphite	263 at 0.1 C	80 at 1 C	50	[162]
PIBs	Graphite	~ 232 at 0.5 C	/	50	[163]
PIBs	Graphite	~ 200 at 0.5 C	~ 80 at 2 C	700	[164]
LSBs	Hollow nitrogen-doped carbon nanospheres	1286 at 0.1 C	623 at 5 C	800	[165]
LSBs	Fructose-derived hollow carbon nanospheres	1043 at 0.1 C	483 at 5 C	200	[166]
LSBs	N-doped porous hollow carbon nanosphere	1224 at 0.2 C	720 at 5 C	500	[167]
LSBs	Hollow N-doped carbon polyhedrons	737.1 at 0.2 C	501.3 at 1 C	500	[168]
LSBs	Hollow carbon nanofibers	1180 at 0.2 C	820 at 1 C	300	[169]
LSBs	Hollow carbon nanofibers	1170 at 1 C	860 at 4 C	300	[170]
LSBs	Hollow carbon nanofiber arrays	730 at 0.2 C	/	150	[171]
LSBs	N-doped hollow carbon spheres	1249 at 0.1 C	688.4 at 2 C	50	[172]
LSBs	3D hyperbranched hollow carbon nanorod	1378 at 0.1 C	663 at 10 C	500	[173]
LSBs	N-doped hollow carbon nanobowls	1065 at 0.1 C	535 at 4 C	400	[174]
LSBs	Hollow carbon foam	699.2 at 0.1 C	525.4 at 0.5 C	100	[175]
LSBs	Hollow N-doped porous carbon nanoparticles	980 at 0.5 C	500 at 9 C	300	[176]

### Lithium-Ion Batteries

**Fig. 8:**
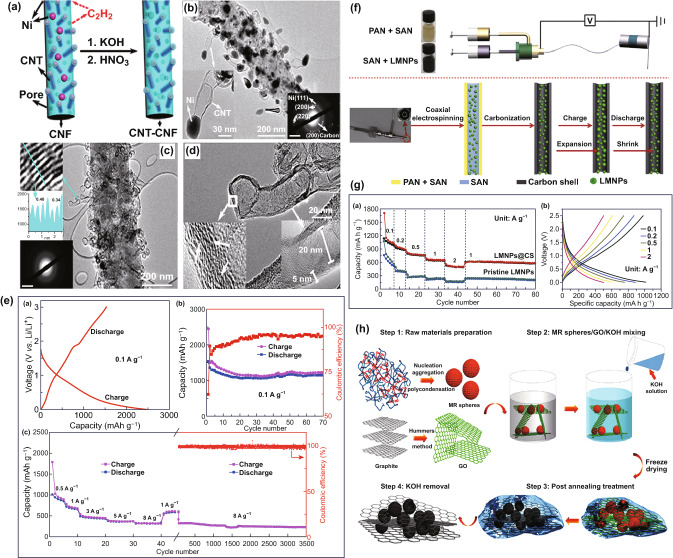
**a** Synthesis schematic, and **c** SAED pattern and TEM image of the activated N-doped hollow CNT–CNF hybrid material. **b** SAED pattern and TEM image of the N-doped CNT–CNF–Ni hybrid material. **d** TEM and HRTEM images of CNT. **e** Electrochemical performance of activated N-doped hollow CNT–CNF hybrid electrode for LIBs. Reproduced with permission from Ref. [127]. Copyright 2013, American Chemical Society. **f** Schematic illustration of the electrospinning steps, and **g** electrochemical performance of the LMNPs@CS electrode. Reproduced with permission from Ref. [135]. Copyright 2019, Elsevier. **h** Synthesis schematic of the DHCSs/RGO composite materials. Reproduced with permission from Ref. [136]. Copyright 2018, American Chemical Society

Lithium-ion batteries (LIBs) have been dominating the power market for portable electronics, and now they are quickly moving into hybrid vehicles and electric transport systems. Graphite anode has been widely used in commercial LIBs, owing to the large specific capacity (372 mAh g^−1^), mechanical robustness, and long cycle stability. However, its poor Li^+^ diffusion kinetics and thick solid electrolyte interphase (SEI) often lead to an inferior rate capability. Up to now, there have been a large number of studies on using nanohollow carbon as the electrode materials for LIBs. For instance, Goodenough and co-works proposed an in situ chemical deposition strategy to grow CNT on the surface of the N-doped CNFs, where C_2_H_2_ as the carbon source and Ni particles as the catalyst, yielding an activated N-doped hollow CNT–CNF hybrid materials (Fig. 8a) [127], in which Ni nanoparticles were encapsulated in graphitic carbon with a thickness of ~ 5 nm (Fig. 8b). They possessed pores and hollow carbon nanoparticles, and defects were present in the wall of hollow structure (Fig. 8c, d). As shown in Fig. 8e, such material gave a reversible capacity of about 1150 mAh g^−1^ at 0.1 A g^−1^. At the high current density of 8 A g^−1^, its capacity of ~ 320 mAh g^−1^ fades less than 20% after 3500 cycles. There are a number of reports on the synthesis of nanohollow carbon materials by template method used for LIBs [128–132, 177, 178]. For example, N-doped mesoporous carbon hollow spheres (N-MCHSs) were synthesized by using mesoporous silica hollow spheres as the template and PDA as carbon precursor, reported by Chu et al. [133]. They displayed a sponge-like mesoporous shell and showed a high specific surface area of 411.6 m^2^ g^−1^. Yu et al. reported graphene-wrapped graphitic hollow carbon spheres (G-graphitic HCS), which were fabricated by iron-catalyzed carbonization of double-coated PS spheres template [134]. They delivered a high initial discharge capacity of 2007 mAh g^−1^ at 0.1 A g^−1^ and remained 92.4% of initial capacity after 100 cycles.

As has been mentioned above, electrospinning is an effective strategy to prepare hollow carbon fibers, which have been applied to LIBs [124, 179, 180]. Chen et al. synthesized a self-healing core fiber with the liquid metal nanoparticles, which were encapsulated with hollow carbon (LMNPs@CS) by the co-axial electrospinning and then carbonization (Figure 8f) [135]. The LMNPs@CS fiber was used as a freestanding anode and showed an impressive rate capability (499 mAh g^−1^ at 2 A^−1^) and excellent cycle stability (552 mAh g^−1^ after 1500 cycles) (Fig. 8g). Yu et al. also proposed a co-axial electrospinning pathway to make hollow carbon nanofibers (HCNFs) as anode materials for LIBs, where the styrene-co-acrylonitrile is the core and poly(acrylonitrile) is the shell solutions, together with a subsequent thermal treatment process [137]. Recently, Lee and co-workers presented a method involving urea coating over the electrospun PAN fibers before the carbonization process [138]. The obtained hollow carbon nanofibers exhibit a significant change in porous structure, which demonstrates a high specific capacity of 520 mAh g^−1^ at 1 C current density when used as the anode for LIBs. Li and co-workers presented a freezing-assisted strategy to achieve N and O co-doped hollow carbon spheres (DHCSs/RGO) composite material (Fig. 8h) [136]. They were coated with thin RGO nanosheets to form a homogeneous 3D porous network architecture. Owing to the hierarchical porous structure and high-level of heteroatom doping, the DHCSs/RGO electrode showed excellent electrochemical performance for LIBs, including a high reversible capacity (1395 mAh g^−1^ at 0.1 A g^−1^), outstanding rate capability (606 mAh g^−1^ at 5 A g^−1^), and long cycle life (600 cycles). Other strategies, such as directly carbonized [181–183], self-assembled [184–186], and microwave-assisted [187], have been employed to prepare nanohollow carbon materials for application in LIBs.

### Sodium-Ion Batteries

**Fig. 9:**
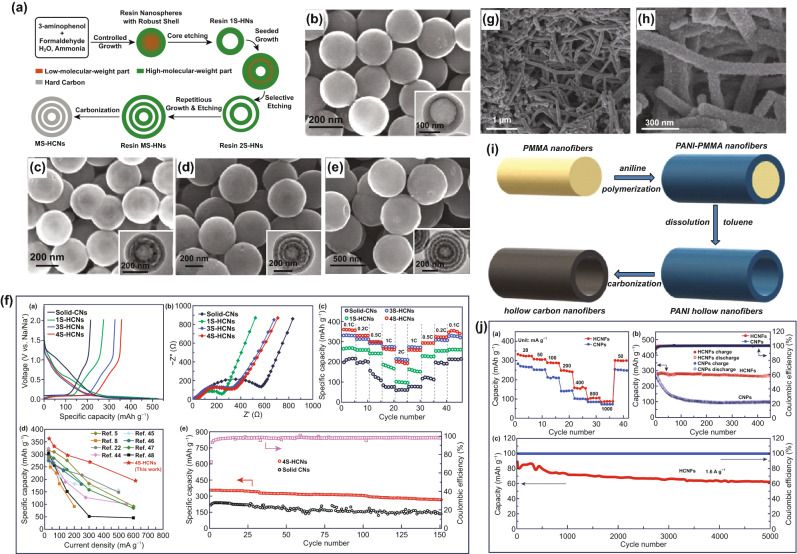
**a** Schematic illustration of the synthesis process for MS-NHCM. SEM and TEM images of **b** 1S-NHCM, **c** 2S-NHCM, **d** 3S-NHCM, and **e** 4S-NHCM samples. **f** Electrochemical performances of all NHCM electrodes for SIBs. Reproduced with permission from Ref. [142]. Copyright 2018, Wiley–VCH. **g**, **h** SEM images of HCNWs. Reproduced with permission from Ref. [147]. Copyright 2012, American Chemical Society. **i** Schematic of the synthesis route of HCNFs. **j** Electrochemical performances of CNPs and HCNFs electrodes for SIBs. Reproduced with permission from Ref. [148]. Copyright 2019, Royal Society of Chemistry

As a low cost and sustainable alternative to LIBs, sodium-ion batteries (SIBs) have been extensively studied over the past few years. The radius of Na^+^ is much larger than that of Li^+^ (1.5 times), often leading to slow intercalation kinetics and poor performances. As an electrode material, hollow carbon nanostructure can provide more space for Na^+^ intercalation and low energy barrier, thus improving the electrochemical kinetics and overall performances. For example, Wan and co-workers proposed a controllable structure engineering to prepare multi-shelled hollow hard carbon nanospheres (MS-NHCMs), which were derived from the hollow resin nanospheres with 3-aminophenol (3-AP) and formaldehyde (3-AF) as the precursors (Fig. 9a) [142]. In particular, single-shelled NHCM (1S-NHCM), double-shelled NHCM (2S-NHCM), three-shelled NHCM (3S-NHCM), and even four-shelled NHCM (4S-NHCM) could be realized through accurate control of the shape parameters (Fig. 9b–e). These NHCM electrodes can deliver better electrochemical performance compared with other carbon-based materials for SIBs. For example, the 4S-NHCM showed a high specific capacity of 360 mAh g^−1^, good rate capability (200 mAh g^−1^ at 2 C), and cycle stability for 150 cycles (Fig. 9f). There are other studies on hollow carbon microspheres, reported for SIBs [143, 144, 188–190]. For example, Dong et al. used the polymethyl methacrylate (PMMA) spheres templates to synthesize S/N-co-doped hollow carbon spheres (SN-HCSs), which showed excellent rate capability (110 mAh g^−1^ at 10 A g^−1^) and cycling performance (2000 cycles) [145]. N-containing hollow carbon microspheres (N-HCSs), made by using modified SiO_2_ as the template and RF the carbon precursor, displayed a reversible capacity of 114 mAh g^−1^ at a high current density of 10 A g^−1^, and long-term cycle stability (> 1200 cycles) [146].

Hollow carbon nanowires and nanofibers have also been used as electrode materials for SIBs [191–194]. For example, Cao and co-workers fabricated hollow carbon nanowires (HCNWs) via pyrolyzation of the hollow polyaniline nanowire precursors [147]. The HCNWs showed a tube-like structure with an outer diameter of ~ 150 nm and an inner diameter of 20–40 nm (Fig. 9g, h). They exhibited a high reversible specific capacity of 251 mAh g^−1^, when used as electrodes in SIBs. Besides, hollow carbon nanofibers (HCNFs) have been prepared by a sacrifice template (PMMA), followed by pyrolyzation of the polyaniline hollow nanofiber precursors (Fig. 9i) [148], showing a superior electrochemical performance, when used as anode materials in SIBs (Fig. 9j), *e.g.*, a high reversible capacity of 326 mAh g^−1^ and good capacity retention of 70% after 5000 cycles. Liang and Yang et al. synthesized N/P co-doped hollow carbon nanofibers and N/S dual-doped hollow carbon fibers, which were employed as the anodes in SIBs [149, 150]. Some resin and biomass-derived hollow carbon materials have been reported for SIBs. For instance, Shen and co-workers synthesized the polyhedral-shaped hollow porous carbon through Ni-ion exchanged resin, together with the carbonization and activation processes [151]. Li et al. used the rape pollen grains as the carbon precursors to acquire 3D hollow reticulate hard carbon through the hydrothermal and high-temperature pretreatment [152]. When employed as the anode in SIBs, this biomass-derived hollow carbon realized an outstanding capacity retention of 90% after 1000 cycles.

### Potassium-Ion Batteries

**Fig. 10:**
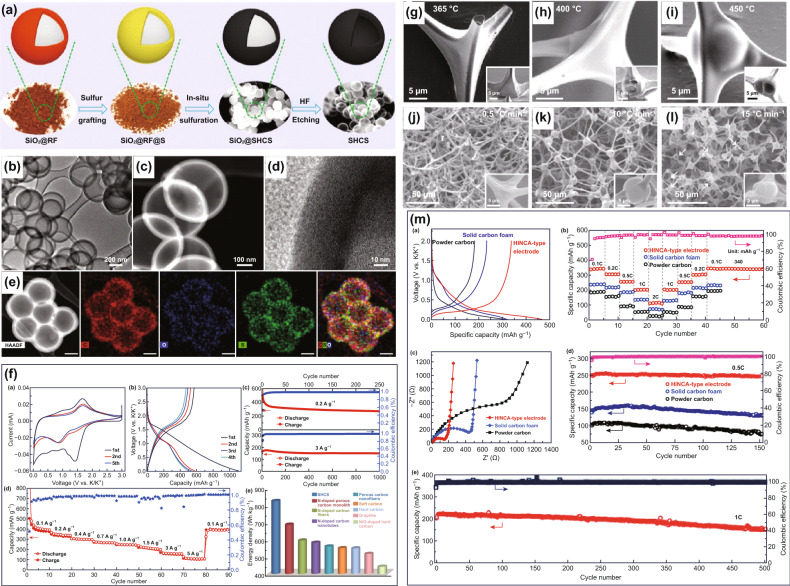
**a** Illustration of the synthesis route of SHCS. **b**, **c** TEM images, **d** HRTEM image, and **e** HAADF image and elements mapping of SHCS. **f** Electrochemical performance of SHCS electrodes for PIBs. Reproduced with permission from Ref. [156]. Copyright 2019, Wiley–VCH. SEM images of morphological change with temperatures of **g** 365, **h** 400, and **i** 450 °C. SEM images of the carbon products with heating temperature rates of **j** 0.5, **k** 10, and **l** 15 °C min^−1^. **m** Electrochemical performance of the HINCA-type, solid carbon foam, and carbon powder electrodes for PIBs. Reproduced with permission from Ref. [159]. Copyright 2018, American Chemical Society

Recently, potassium-ion batteries (PIBs) have attracted attention as promising alternatives to LIBs and SIBs. In particular, PIBs are expected to offer a higher operation potential in wider voltage range, compared to SIBs. Al-K intermetallic compounds would not be formed during charge–discharge processes, and thus, the low-cost Al foil can be used as the current collector for both cathode and anode electrodes in PIBs. Up to now, there have been reports on hollow carbon materials that are used as active materials for PIBs. For instance, Mitlin and co-workers proposed the sulfur-grafted hollow carbon spheres (SHCS) as anode materials for PIBs [156]. These SHCS were synthesized via a sulfuration strategy, with RF resin as the carbon source, sulfur powder as the sulfur source, and silica sphere as the hard template (Fig. 10a). They showed a monodispersed hollow sphere nanostructure, with a rather uniform diameter (~ 400 nm) and thickness (~ 40 nm) (Fig. 10b, c). There is an amorphous structure observed, and the C, O, S elements were seen to evenly distribute in the carbon skeleton (Fig. 10d, e). Benefiting from a high amount of sulfur-grafted (38 wt%) and hollow structure, the SHCS electrode displayed an impressive electrochemical performance for PIBs (Fig. 10f). They can deliver an ultrahigh reversible capacity of 581 mAh g^−1^ and an excellent capacity retention of 93% after 1000 cycles. Activated hollow carbon nanospheres (AHCSs) have been synthesized and used as anode materials for PIBs, with RF as the carbon source and SiO_2_ as the template [157]. Recently, Chen et al. utilized PDA as both the carbon and N-doping sources and silica spheres as the template in preparing N-doped hollow carbon nanospheres (N-NHCMs), which gave a high reversible capacity of 154 mAh g^−1^ (1.0 A g^−1^ current density) over 2500 cycles, when employed as the anode electrode in PIBs [158].

Taking the integrity and effectiveness of structures into consideration, the cross-linked hollow structure can not only improve the structural stability, but also increase the electron and ion transfer rate of materials. In view of this, Wan and co-workers have proposed a structural engineering strategy to design carbon materials such that they display a hollow interconnected neuron-like carbon architecture (HINCA) [159]. Such a structure was prepared by high-temperature carbonization of a commercial RF resin foam. There was structural transformation, as shown in Fig. 10g–i, where the SEM images of the HINCA-type products showed a morphological change at different treatment temperatures of 365, 400, and 450 °C. The inner cavity increased, and the wall thickness shrunk with the increase in temperatures. The effect of heating rate (Fig. 10j–l) did not appear to affect the interconnected architecture, as shown for those at different heating rates of 0.5, 10, and 15 °C min^−1^. When the carbonization temperature was 1300 °C, the as-fabricated HINCA-type electrode shows superior electrochemical performance, compared with the solid carbon foam and power carbon electrode for PIBs (Fig. 10m). In particular, it delivered a reversible capacity of 340 mAh g^−1^ at 0.1 C and maintained 72.1% of capacity after 500 cycles. With a similar structural design, Qin and co-workers obtained hollow multihole carbon bowls (CHMBs) by hydrothermal carbonization coupled with a soft template (emulsion) strategy [160]. The as-fabricated CHMBs electrodes showed a high specific capacity of 304 mAh g^−1^ at 0.1 A g^−1^ and ultra-long cycling stability (1000 cycles). More recently, Mai et al. synthesized polycrystalline soft carbon semi-hollow microrods, which gave an impressive reversible capacity of 314 mAh g^−1^, when used as an anode for PIBs [161].

### Lithium–Sulfur Batteries

Lithium–sulfur batteries (LSBs) are among the most promising next-generation batteries systems, owing to their high theoretical specific capacity (1675 mAh g^−1^), where the sulfur cathode also offers other advantages, including an abundant source, low cost, and environmental friendliness. However, LSBs face challenges in practical sense, such as a low electronic conductivity and large volume expansion of the sulfur cathode, the dissolution and shuttle effect of polysulfide, leading to a rapid capacity fading and poor cycle stability, together with low Coulombic efficiency. To solve some of these problems, NHCMs have been employed as host materials for sulfur cathode, owing to their good electronic conductivity and tailored shell structures. Indeed, several NHCMs have been reported as host materials for application in LSBs, including those hollow carbon nanospheres, hollow carbon nanotubes/nanofibers, hollow carbon nanobowls, hollow carbon foams, and hollow carbon polyhedrons [6, 10, 11].

**Fig. 11:**
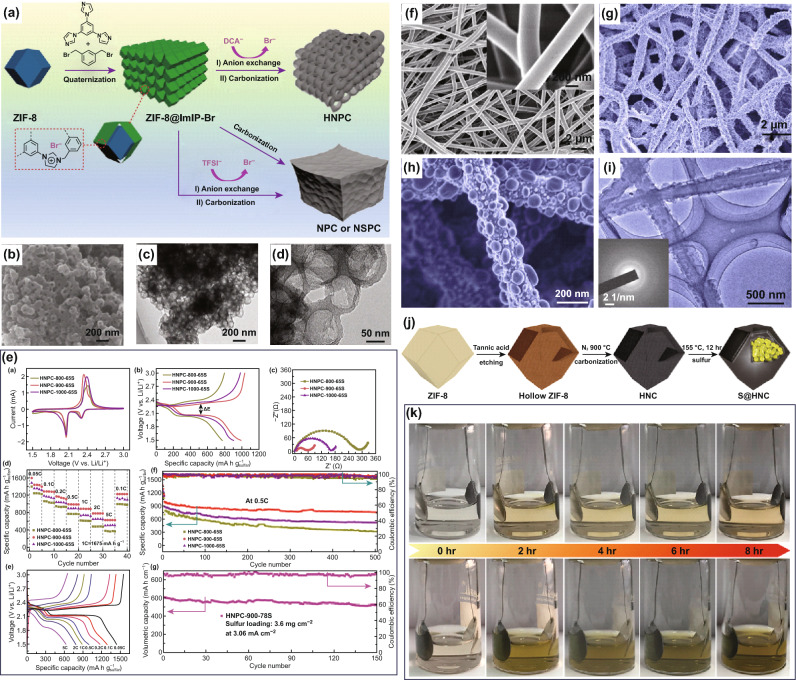
**a** Schematic illustration of the synthesis route for HNPC. **b** SEM image and **c**, **d** TEM images of the HNPC-900. **e** Electrochemical performance of HNPC electrodes for LSBs. Reproduced with permission from Ref. [165]. Copyright 2019, Wiley–VCH. **f** SEM images of the electrospun PVA-LiN_3_ nanofibers. **g**, **h** SEM images of PCNFs after calcination. **i** TEM image of the CHNBs@PCNFs. Reproduced with permission from Ref. [206]. Copyright 2018, Elsevier. **j** Schematic illustration of the synthesis process for S@HNC hybrid. **k** Digital images of S@HNC and S-NC electrodes during the discharge process for LSBs. Reproduced with permission from Ref. [168]. Copyright 2019, Cell Publishing Group

Among them, hollow carbon nanospheres are the most commonly employed host materials for LSBs, owing to the large cavity that can effectively accommodate the large volume expansion and shuttle effect of polysulfides during the charge–discharge processes [195–205]. For instance, Wang et al. synthesized the hollow nitrogen-doped carbon (HNPC) by anion exchange and subsequent pyrolysis, using rhombic dodecahedral ZIF-8 as the core and imidazolium-based ionic polymers as the shell layer (Fig. 11a) [165]. The HNPC-900 (carbonization at 900 °C) thus made showed a 3D hollow nanosphere network (Fig. 11b–d), which was a desirable structure for sulfur loading. The HNPC-900 electrode delivered a superior electrochemical performance compared with HNPC-800 and HNPC-1000 in LSBs (Fig. 11e), including an excellent reversible capacity of 562 mAh g^−1^ at 2 C and prolonged cycle stability of 800 cycles. Recently, Shao et al. presented a dual template-assisted strategy to prepare hollow carbon nanospheres (HCS) with diameters of ~ 300 nm [166], which was then employed as sulfur host materials for LSBs. It exhibited a high reversible capacity of 585 mAh g^−1^ at 2 C and long cycle stability of 600 cycles. Notably, the hollow carbon nanospheres were also reported to decorate separators in LSBs. For example, Zhang and co-workers designed the separator coated with the N-doped porous hollow carbon nanosphere (NHC) to improve the utilization of sulfur cathode and suppress the shuttle effect of polysulfides [167]. They found that the NHC decorated separator could achieve superior electrochemical performance, including a high reversible capacity of 720 mAh g^−1^ at 5 C and an outstanding cycle lifespan of 500 cycles.

In addition to hollow carbon nanospheres, hollow carbon nanotubes/nanofibers have also been reported as favorable host materials for LSBs [169–172]. For example, Yu and co-workers employed an electrospinning strategy to fabricate hollow carbon nanobubbles on porous carbon nanofibers (CHNBs@PCNFS) [206], which are shown in Fig. 11f–i. In the electrospun PVA-LiN_3_ nanofibers, the porous hollow carbon nanobubbles were well attached to the surface of hollow carbon nanofibers. Han et al. developed hollow N-doped carbon polyhedrons (HNC) as the host of the sulfur cathode, which was fabricated via chemical etching and carbonization process (Fig. 11j) [168]. Such electrode realized a high sulfur loading of ~ 72 wt% and an impressive reversible capacity of 501.3 mAh g^−1^ at 1 C, together with a long cycle life (500 cycles). The HNC also showed a strong chemical interaction with polysulfides by the visualized measurement of S@HNC and S-NC electrodes during the discharge process (Fig. 11k). Other hollow carbon nanostructures, such as hollow carbon nanorods [173], hollow carbon nanobowls [174], hollow carbon foams [175], and hollow carbon nanoparticles [176], have been also employed as host materials for the sulfur cathode.

## Conclusion and Outlook

In summary, we have reviewed the ongoing progresses of exploring nanohollow carbon materials (NHCMs), including their synthesis strategies, different types of morphologies, and performance in several types of rechargeable batteries. For each of the morphologies of hollow nanospheres, nanopolyhedrons, and nanofibers, a proper tuning in the key structural features by processing controls, NHCMs show great potential as the electrode materials in rechargeable batteries, including LIBs, SIBs, PIBs, and LSBs. In addition to being electrodes by themselves, NHCMs also act as an efficient supporting substrate for other active materials, improving the loading, overall electronic conductivity, and mechanical stability, leading to faster reaction kinetics, better performance, and long cycle ability [207]. Although considerable progress has been made so far, in almost all aspects, attempted by various strategies as described in this overview, there are several unsolved issues and challenges, which should be addressed further, especially for developing large-scale production at low cost, and application in energy storage.

For NHCMs fabrication, various synthesis strategies (hard templating, soft templating, template-free, MOF-derived, and electrospinning) have been developed. In particular, the hard template methods are currently the most widely used, giving rise to various desired key structure features. However, the synthesis processes of hard templates are multi-steps and time-consuming, owing to the pre-making and removal of templates, together with the use of high-cost carbon precursors. The rather tedious, high-cost, and low-yield multi-steps are the apparent disadvantages. On the other hand, the various soft template methods are relatively simple and can be applied on a large scale by eliminating the template etching process. However, the morphologies and key features of the as-fabricated NHCMs are usually difficult to control. The newly emerged template-free approaches give opportunities to synthesize NHCMs, by the combination of facile processing, low cost, and generally high uniformity. For example, aerosol spraying is a typical template-free approach, in which the high production yield, cost-effectiveness, and continuous process enable a better control in the desired morphologies and compositions. Nevertheless, most of the template-free methods are relatively immature up to now. Therefore, further development of the existing template-free methods and devising new ones shall be pursued for NHCMs, especially those leading to the desired hollow structures. In this connection, metal–organic frameworks derived nanohollow carbon polyhedrons and electrospun nanohollow carbon nanofibers are among the exciting ongoing research and development. There is no doubt that more effective strategies will appear in the coming years, and they will be explored for applications in energy storage and other related devices.

For NHCMs applications, the first key issue is the relatively low volume energy density that can be derived, because of their low tap density. Therefore, new hollow nanostructures shall be designed such that the cavities in NHCMs will be used as much as possible. For example, the development of multi-shelled and yolk–shell structures can be a suitable approach to improve the volumetric energy density, with other advantages. Secondly, the storage mechanisms of NHCMs when employed as the electrode materials for rechargeable batteries need to be understood thoroughly. In addition, the key structural features such as the inter-layer spacing of NHCMs shall be different for the respective Li-ion, Na-ion, and K-ion batteries, owing to their different ionic radii and diffusion rates. Also, the specific energy storage (Li^+^, Na^+^, K^+^) mechanisms would be different among them. For example, for rechargeable LSBs, the HNCS can be used as a host material to effectively fill the sulfur cathode and suppress the shuttle effect of polysulfides. Therefore, the corresponding energy storage mechanisms need to be properly investigated and understood with the help of some advanced in situ characterizations, such as in situ XRD, SEM, TEM, AFM, together with some theoretical calculations, including the density functional theory (DFT), and molecular dynamics (MD). Thirdly, heteroatom doping (N, O, B, S, P, etc.) has been widely applied in NHCMs to improve their electrochemical performance and reaction kinetics. The heteroatom types, corresponding doping levels, and their impact on the structures and properties of NHCMs need to be further explored. Fourthly, the NHCMs electrodes often exhibit certain initial irreversibility, which would severely consume ions from the electrolyte and cathodes, leading to low Coulombic efficiency and poor cycle lifespan. Thus, it would be of value to develop an appropriate ion pre-embedding technique for the full-battery systems. We hope that this review can provide an avenue for better understanding of the design and synthesis of nanohollow carbon materials and stimulate greater interests and efforts toward their functionalization and applications.
